# Low levels of circulating methylated *IRX3* are related to worse outcome after transcatheter aortic valve implantation in patients with severe aortic stenosis

**DOI:** 10.1186/s13148-023-01561-2

**Published:** 2023-09-11

**Authors:** Leon Kanwischer, Xingbo Xu, Afifa Binta Saifuddin, Sabine Maamari, Xiaoying Tan, Fouzi Alnour, Björn Tampe, Thomas Meyer, Michael Zeisberg, Gerd Hasenfuss, Miriam Puls, Elisabeth M. Zeisberg

**Affiliations:** 1 Department of Cardiology and Pneumology, University Medical Center Göttingen, Georg-August-University, Robert-Koch-Str. 40, 37075 Göttingen, Germany; 2 Department of Nephrology and Rheumatology, University Medical Center Göttingen, Georg-August-University, Göttingen, Germany; 3 Department of Psychosomatic Medicine and Psychotherapy, University Medical Center Göttingen, Georg-August-University, Göttingen, Germany; 4 DZHK German Center for Cardiovascular Research, Partner Site Göttingen, Göttingen, Germany

**Keywords:** Aortic stenosis, IRX3, Cardiac fibrosis, Epigenetics, DNA methylation, Biomarker

## Abstract

**Background:**

Aortic stenosis (AS) is one of the most common cardiac diseases and major cause of morbidity and mortality in the elderly. Transcatheter aortic valve implantation (TAVI) is performed in such patients with symptomatic severe AS and reduces mortality for the majority of these patients. However, a significant percentage dies within the first two years after TAVI, such that there is an interest to identify parameters, which predict outcome and could guide pre-TAVI patient selection. High levels of cardiac fibrosis have been identified as such independent predictor of cardiovascular mortality after TAVI. Promoter hypermethylation commonly leads to gene downregulation, and the Iroquois homeobox 3 (IRX3) gene was identified in a genome-wide transcriptome and methylome to be hypermethylated and downregulated in AS patients. In a well-described cohort of 100 TAVI patients in which cardiac fibrosis levels were quantified histologically in cardiac biopsies, and which had a follow-up of up to two years, we investigated if circulating methylated DNA of *IRX3* in the peripheral blood is associated with cardiac fibrosis and/or mortality in AS patients undergoing TAVI and thus could serve as a biomarker to add information on outcome after TAVI.

**Results:**

Patients with high levels of methylation in circulating *IRX3* show a significantly increased survival as compared to patients with low levels of *IRX3* methylation indicating that high peripheral *IRX3* methylation is associated with an improved outcome. In the multivariable setting, peripheral *IRX3* methylation acts as an independent predictor of all-cause mortality. While there is no significant correlation of levels of *IRX3* methylation with cardiac death, there is a significant but very weak inverse correlation between circulating *IRX3* promoter methylation level and the amount of cardiac fibrosis. Higher levels of peripheral *IRX3* methylation further correlated with decreased cardiac IRX3 expression and vice versa.

**Conclusions:**

High levels of *IRX3* methylation in the blood of AS patients at the time of TAVI are associated with better overall survival after TAVI and at least partially reflect myocardial IRX3 expression. Circulating methylated *IRX3* might aid as a potential biomarker to help guide both pre-TAVI patient selection and post-TAVI monitoring.

**Graphical abstract:**

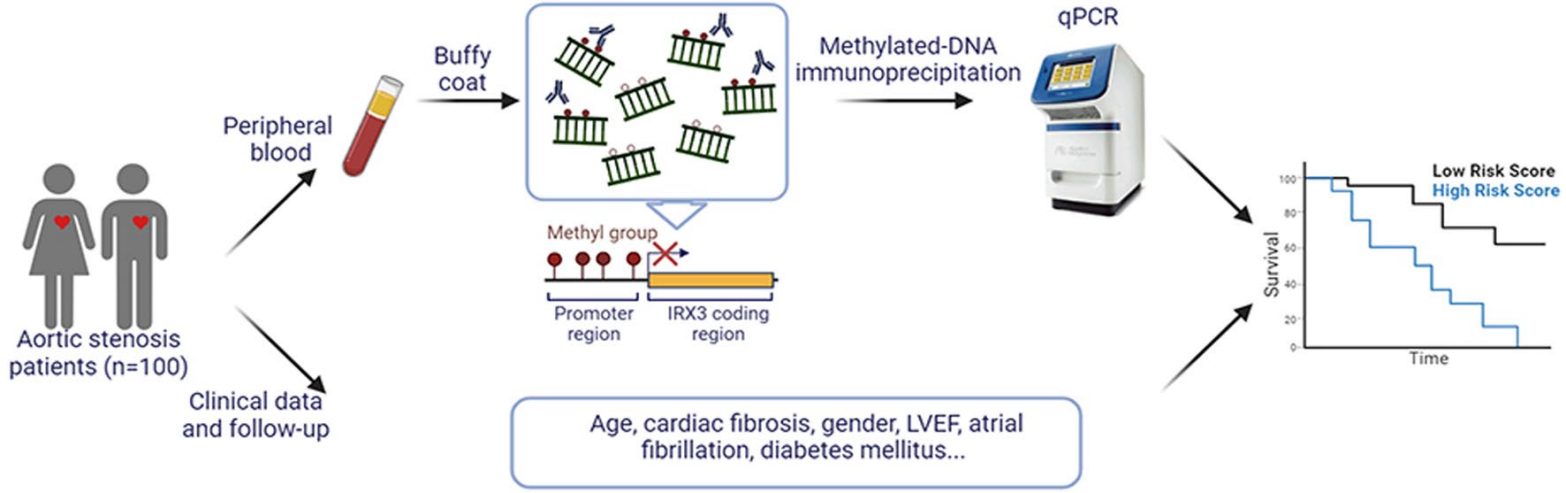

## Background

Aortic stenosis (AS) is a common cardiac disease in the elderly with a prevalence as high as 9.8% in the population older than 80 years [1]. In the advanced stage of AS, the narrowed valve leads to pressure overload of the left ventricular system and reduced left ventricular ejection fraction (LVEF) thus finally to heart failure [2]. Current guidelines recommend transcatheter aortic valve implantation (TAVI) as the intervention mode for symptomatic AS patients with increased age and high surgical risk, whereas evidence for intervention to improve outcomes in asymptomatic severe AS is less strong [3]. Thus, the appropriate timing of intervention and risk stratification of AS patients is highly important.

Biomarkers are essential and well established in the diagnosis of cardiovascular diseases, such as heart failure or myocardial infarction (MI) [4, 5]. However, although conventional biomarkers, such as B-type natriuretic peptide (BNP) levels, show associations with mortality in AS patients, they are not fully established and play a minor role in diagnostic guidelines [2, 6]. At the same time, insight into the impact of different epigenetic mechanisms on the pathogenesis of AS is increasing and their potential utility as biomarkers has recently been described [7]. Also, current research showed the impact of cardiac fibrosis (CF) on increased mortality after TAVI, so non-invasive assessment of CF by cardiovascular magnetic resonance imaging (CMR) can be considered as a future biomarker for risk stratification in AS patients [8, 9]. However, these approaches are not implemented into clinical routine diagnostics of AS and independent and specific biomarkers to inform on individual risk of AS patients are still needed.

Epigenetics contribute to the development and pathogenesis of numerous diseases [10]. In this respect, the process of DNA methylation leads to silencing of gene expression and is catalyzed by DNA methyltransferases, but can be reactivated via hydroxymethylation catalyzed by TET (ten eleven translocation) enzymes [11–13]. This offers the opportunity for a possible therapeutic approach by methylating and demethylating drugs, which have already been investigated in preclinical studies and even some clinical trials for cardiovascular diseases [14]. While the role of DNA methylation has not yet been thoroughly investigated in the pathogenesis of AS, first results showed altered methylation of distinguished genes in both congenital and acquired AS [15, 16].

Epigenetic alterations in circulating DNA were first reported to reflect tumor DNA and might aid as a biomarker to inform on tumor progression [17]. The release of DNA into the peripheral blood occurs by necrosis, apoptosis and active secretion [18, 19]. It could also be observed under inflammatory conditions, raising the possibility to ensue in the inflammatory process in the pathogenesis of AS as well [20]. For other cardiovascular diseases, including heart failure, elevated levels of specific circulating DNA have been described and discussed as potential biomarkers [21, 22]. Moreover, in the search for new epigenetic biomarkers of cardiovascular diseases, high hopes are pinned on the application of powerful research approaches such as network medicine and artificial intelligence, linking molecular findings to clinical parameters and imaging tools [23, 24].

A genome-wide transcriptome and methylome comparison (MeDIP-seq) between cardiac tissue of AS patients and healthy controls by our group identified the Iroquois homeobox 3 (IRX3) protein to be simultaneously downregulated in gene expression and hypermethylated in the promoter region. IRX3 is known to influence embryonic neural development in the ventral neural tube and motor neurons [25, 26]. Recent findings revealed its role in the metabolic system, where deficiency of IRX3 was protective in a mouse model of obesity [27]. The role of IRX3 in the cardiovascular field has not yet been fully identified. It could be shown that a knockout of IRX3 leads to malformations during embryonic and postnatal development, especially in the outflow area and the ventricular conduction system [28, 29]. Even in the adult heart, mutations of *IRX3* are associated with idiopathic ventricular fibrillation, which presents a high risk of sudden cardiac death [30].

Here, we aimed to assess if circulating methylated *IRX3* is associated with cardiac fibrosis and/or survival in AS patients after TAVI. We first assessed the promoter activity of the differentially methylated region (DMR) identified from the MeDIP-seq analysis by a promoter activity assay and confirmed that there is reduced gene activity due to hypermethylation. In a random subset of 10 out of the 88 patients who received a cardiac biopsy, we next analyzed *IRX3* expression in cardiac biopsies in addition to circulating methylated DNA fragments. This analysis demonstrated that patients with low circulating *IRX3* promoter methylation had a higher *IRX3* expression in the heart, suggesting that circulating *IRX3* promoter methylation at least to some extent represents *IRX3* expression in the heart. Finally, we examined the circulating *IRX3* promoter methylation and its association to cardiac fibrosis and to mortality in 100 AS patients who underwent TAVI and were followed up for two years after TAVI. We found that a lower promoter methylation of *IRX3* was associated with a significantly higher all-cause but not cardiovascular mortality within two years after TAVI in both univariable and multivariable analyses, suggesting circulating methylated *IRX3* DNA as a novel prognostic biomarker in patients with AS.

## Results

### Promoter hypermethylation causes downregulation of gene expression in vitro

**Fig. 1:**
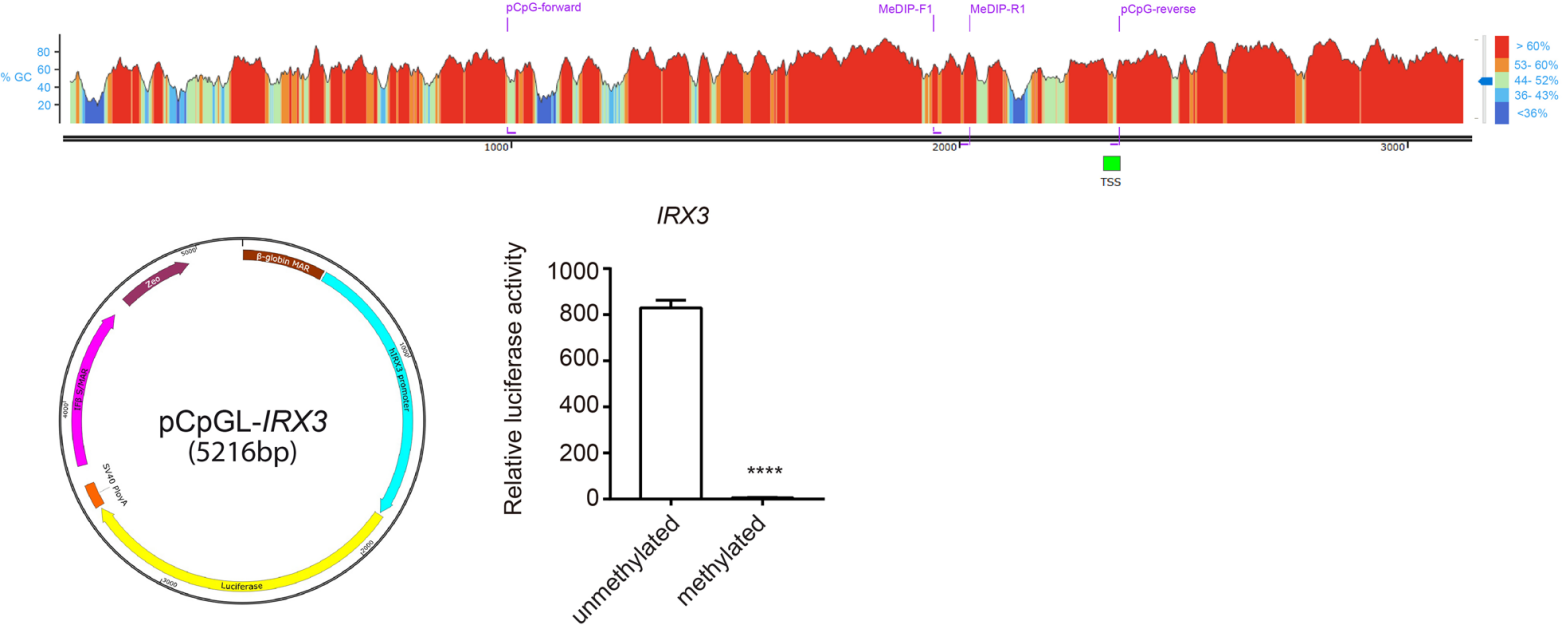
Promoter hypermethylation of *IRX3* causes reduction of gene expression in vitro. GC content summary of *IRX3* promoter region with the locations for the pCpGL cloning primers and MeDIP primers. Plasmid map of pCpGL-*IRX3* (5216 bp) with *IRX3* promoter sequence (1364 bp) inserted before the firefly luciferase section. Quantification of normalized luciferase activity showed a 143.4-fold reduction for the methylated construct as compared to the unmethylated control. Red color indicates a high amount of GC content, whereas blue color represents low GC content. One-way ANOVA was used for multiple group comparison. Relative luciferase activity and associated error bars represent mean ± SEM, *n* ≥ 3, **** *p* < 0.0001. Plasmid map was created using the SnapGene software (version 4.2.4), Insightful Science, San Diego, CA, USA. TSS: transcription start site

In order to assess if promoter methylation of *IRX3* leads to its downregulation, we performed a promoter activity assay. The DNA sequence of the DMR of *IRX3* as identified from the MeDIP-seq analysis (from − 1330 bp to 34 bp) was inserted into the pCpGL-basic vector containing a firefly luciferase encoding region. The construct was then subjected to an in vitro methylation assay, and the methylated construct was delivered into HEK-293 cells by transfection. The luciferase activity of the methylated pCpGL-*IRX3* was significantly reduced as compared to the unmethylated control (Fig. 1). This result suggests that the DMR, which was identified from the MeDIP-seq assay, has a strong promoter activity for *IRX3* gene. Therefore, the MeDIP-qPCR (methylated DNA immunoprecipitation-real-time quantitative polymerase chain reaction) assay was established within this region.

### *IRX3* expression and connection to the promoter methylation in human cardiac biopsies

This study includes 100 study participants who underwent TAVI in the Department of Cardiology and Pneumology at the University Medical Center Göttingen (UMG). Blood samples were available from all patients at the time of TAVI. Biopsies, in which cardiac fibrosis was quantified, were taken during the TAVI procedure and were available from 88 of these patients. To investigate the connection between circulating *IRX3* promoter methylation and cardiac *IRX3* expression, we isolated the DNA from the buffy coat of the blood samples and performed a MeDIP-qPCR assay. This allowed us to divide the study cohort into a group of high (IRX3^5mc−high^) and low (IRX3^5mc−low^) promoter methylation. We used a random subset of five samples of each group for *IRX3* expression analysis by immunohistochemistry staining with IRX3 antibody.

**Fig. 2:**
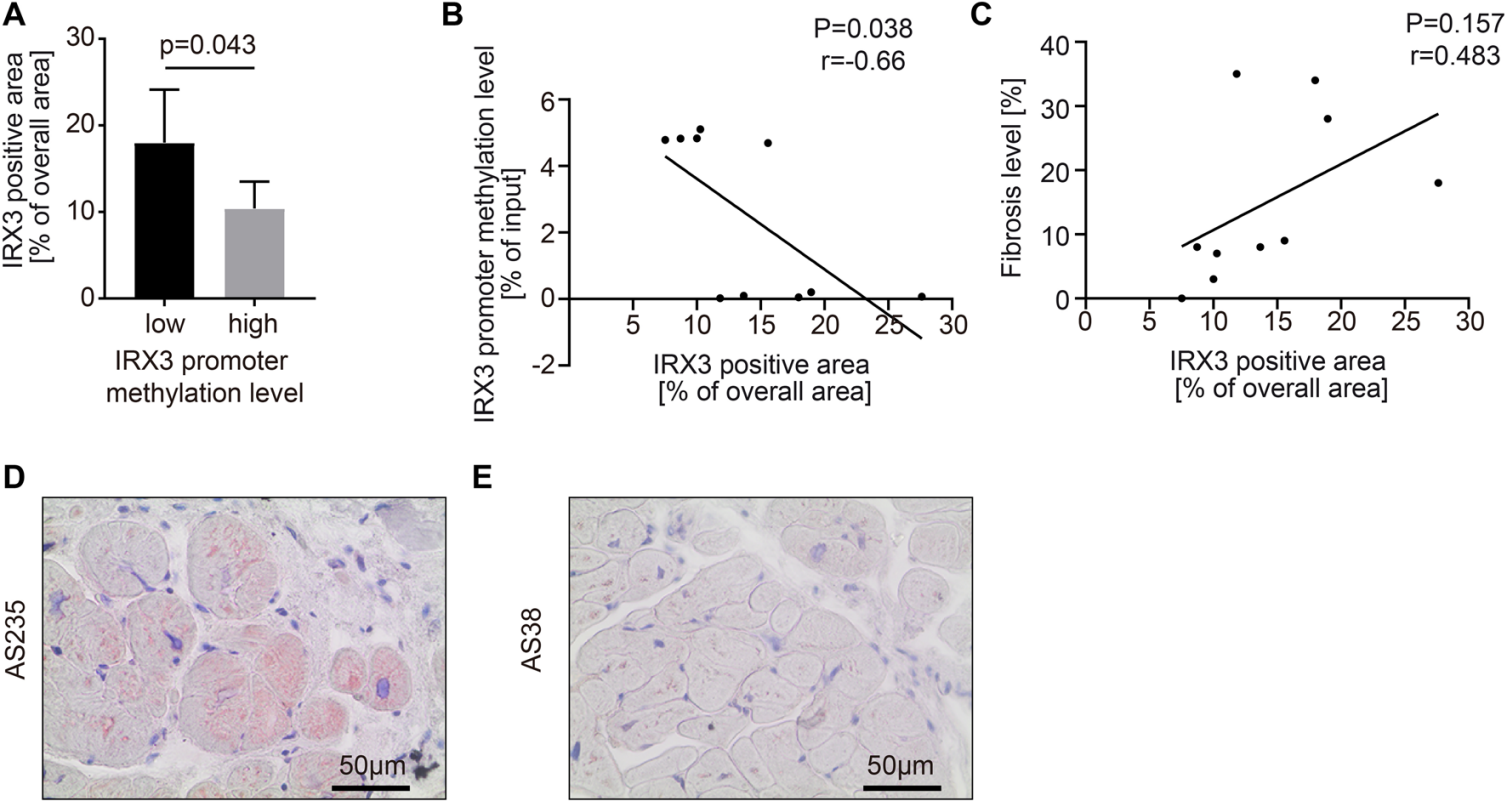
Immunohistochemical staining of IRX3 and its correlation to the circulating promoter methylation level. **A** Quantification of IRX3 positive area. Compared to the group of circulating *IRX3* promoter methylation above the median (gray), the group of circulating *IRX3* promoter methylation below the median (black) showed a higher amount of IRX3 positive stained area. **B** Correlation analysis between circulating *IRX3* promoter methylation and the amount of IRX3 positive stained area revealed a significant inverse correlation (*r* = − 0.66) **C** Correlation analysis between cardiac fibrosis and the amount of IRX3 positive stained area showed no significance for the correlative tendency. **D** Immunohistochemical staining of IRX3 (red color) in a cardiac biopsy of a patient from the low circulating *IRX3* promoter methylation group showing a high amount of IRX3. **E** Immunohistochemical staining of IRX3 (red color) in a cardiac biopsy of a patient from the high circulating *IRX3* promoter methylation group showing a low amount of IRX3. Student’s t-test was used for single comparison. IRX3 positive area and associated error bars represent mean ± SEM, *n* = 5

The group of low *IRX3* promoter methylation exposed a higher mean IRX3 positive area in the cardiac biopsies (IRX3^5mc−low^, 18.0% ± 2.7%; IRX3^5mc−high^, 10.4% ± 1.4%, Fig. 2 A). Additionally, the IRX3 positive area and the *IRX3* promoter methylation showed a significant inverse correlation (*p* = 0.038, Fig. 2 B). However, the IRX3 positive area and the amount of cardiac fibrosis did not show a significant correlation (Fig. 2C). This result implies a possible correlation between cardiac *IRX3* expression and circulating *IRX3* promoter methylation.

### In vivo data of one hundred patients with severe AS

#### Baseline characteristics

The total cohort which was named above contained 41 women and 59 men with a mean age of 78.4 ± 0.6 years, indicating for advanced age. The number of co-morbidities such as coronary heart disease (70%), diabetes mellitus (39%) and atrial fibrillation (41%) was relatively high in this cohort, as well as the average NYHA-class (New York Heart Association) of 2.78 ± 0.08 (Table 1).

**Table 1:** Baseline characteristics depending on all-cause mortality

	Total cohort (*n* = 100)	Death (*n* = 25)	No death (*n* = 75)	*p* value
Methylation level IRX3 (%)	1.74 ± 0.15	1.27 ± 0.20	1.90 ± 0.18	0.026*
Age (years)	78.4 ± 0.6	76.4 ± 1.3	79.1 ± 0.7	0.081
Female sex, n (%)	41	10 (40)	31 (41)	0.907
BMI (kg/m^2^)	28.1 ± 0.5	28.7 ± 1.3	27.9 ± 0.6	0.587
Cardiac fibrosis (%)	17.8 ± 2.2	22.8 ± 4.8	16.0 ± 2.4	0.166
LVEF (%)	51.8 ± 1.6	47.5 ± 3.6	53.4 ± 1.7	0.098
LAVI (ml/m^2^)	49.9 ± 1.6	56.2 ± 4.1	47.9 ± 1.7	0.034*
LVEDD (mm/m^2^)	24.5 ± 0.4	24.6 ± 0.9	24.5 ± 0.5	0.945
LVESD (mm/m^2^)	18.7 ± 0.5	18.8 ± 1.2	18.7 ± 0.6	0.925
LVMI (g/m^2^)	145.0 ± 4.6	150.7 ± 9.5	142.9 ± 5.3	0.457
Vmax (m/s)	3.90 ± 0.07	3.75 ± 0.13	3.95 ± 0.09	0.239
MPG (mmHg)	36.6 ± 1.5	33.3 ± 2.2	37.7 ± 1.9	0.218
AVA (cm^2^)	0.75 ± 0.02	0.71 ± 0.04	0.76 ± 0.02	0.247
Plasma creatinine (mg/dl)	1.24 ± 0.07	1.45 ± 0.17	1.17 ± 0.08	0.102
6MWT (m)	222.6 ± 13.1	167.8 ± 22.1	241.2 ± 15.2	0.014*
STS score	5.25 ± 0.65	5.89 ± 0.96	5.02 ± 0.81	0.556
EuroSCORE II	6.63 ± 0.80	7.79 ± 1.33	6.23 ± 0.98	0.393
NYHA-class	2.78 ± 0.08	3.12 ± 0.11	2.67 ± 0.09	0.002**
AF, n (%)	41	16 (64)	25 (33)	0.007**
CAD, n (%)	70	15 (60)	55 (73)	0.208
Prior CABG, n (%)	12	2 (8)	10 (13)	0.477
Prior MI, n (%)	18	5 (20)	13 (17)	0.764
PAD, n (%)	24	11 (44)	13 (17)	0.007**
DM, n (%)	39	13 (52)	26 (35)	0.124
CRD, n (%)	26	9 (36)	17 (23)	0.188

All continuous variables are presented as mean ± SEM. All categorical variables are presented as absolute and relative frequency

*p < 0.05, **p < 0.01

*6MWT* 6-min walking test; *AF* Atrial fibrillation; *AVA* Aortic valve area; *BMI* Body mass index; *CABG* Coronary artery bypass graft; *CAD* Coronary artery disease; *CRD* Chronic respiratory disease (chronic obstructive pulmonary disease or lung fibrosis); *Death* Patients who died in the period of observation, no further specification of the cause of death; *DM* Diabetes mellitus; *EuroSCORE* European System for Cardiac Operative Risk Evaluation; *IRX3* Iroquois homeobox 3; *LAVI* Left atrial volume index; *LVEDD* Left ventricular end-diastolic diameter normalized to body surface area (BSA); *LVEF* Left ventricular ejection fraction; *LVESD* Left ventricular end-systolic diameter normalized to BSA; *LVMI* Left ventricular mass index; *MI* Myocardial infarction; *MPG* Mean pressure gradient; *No death* Patients who did not die in the period of observation; *NYHA* New York heart association; *PAD* Peripheral artery disease; *STS* Society of Thoracic Surgeons; *Vmax* Aortic jet velocity. *Group comparison* Unpaired Student’s t-test for continuous variables and Pearson’s Chi^2^-test for categorical variables

After identifying the participants who died in the observation period (all-cause mortality), we observed that the 6-min walking test (6MWT) distance was significantly lower in the group of deaths and the mean NYHA-class was significantly higher in this group. LAVI was significantly increased in the group of deaths. The LVEF tended to be reduced in the group of deaths but didn’t reach a level of significance (death, 47.5% ± 3.6%; no death, 53.4% ± 1.7; *p* = 0.098) (Table 1). Additionally, peripheral artery disease and atrial fibrillation as co-morbidities were more common in the group of deaths. Interestingly, the promoter methylation of *IRX3* in the peripheral blood, which was assessed using MeDIP-qPCR assay, was significantly lower in the group of deaths than in the group of survivors (death, 1.27% ± 0.20%; no death, 1.90% ± 0.18%).

Additional analysis of baseline parameters for cardiac deaths revealed differences regarding several cardiac parameters: The amount of cardiac fibrosis was significantly increased in the cardiac deaths (cardiac death, 33.4% ± 6.3%; no cardiac death, 14.8% ± 2.1%). Additionally, the LVEF was significantly reduced in the group of cardiac deaths (Table 2). The peak aortic jet velocity and the mean pressure gradient between the left ventricle and the ascending aorta were significantly lower and the aortic valve area tended to be smaller in the group of cardiac deaths than in the group of no cardiac deaths, representing a high amount of low-gradient AS in the cardiac deaths (Table 2). Interestingly, a very high number of participants in the group of cardiac deaths suffered from diabetes mellitus (cardiac death, 86%; no cardiac death, 31%). In contrast to the comparison depending on all-cause mortality, the promoter methylation of *IRX3* showed no significant difference between cardiac deaths and no cardiac deaths (Table 2).

**Table 2:** Baseline characteristics depending on cardiac mortality

	Total cohort (*n* = 100)	Cardiac death (*n* = 14)	No cardiac death (*n* = 86)	*p* value
Methylation level IRX3 (%)	1.74 ± 0.15	1.37 ± 0.28	1.80 ± 0.16	0.198
Age (years)	78.4 ± 0.6	76.3 ± 1.9	78.7 ± 0.7	0.190
Female sex, n (%)	41	4 (29)	37 (43)	0.308
BMI (kg/m^2^)	28.1 ± 0.5	30.6 ± 2.0	27.7 ± 0.5	0.196
Cardiac fibrosis (%)	17.8 ± 2.2	33.4 ± 6.3	14.8 ± 2.1	0.001**
LVEF (%)	51.8 ± 1.6	42.0 ± 4.7	53.8 ± 1.6	0.030*
LAVI (ml/m^2^)	49.9 ± 1.6	52.4 ± 4.2	49.4 ± 1.8	0.528
LVEDD (mm/m^2^)	24.5 ± 0.4	25.2 ± 1.3	24.4 ± 0.4	0.536
LVESD (mm/m^2^)	18.7 ± 0.5	18.4 ± 0.5	20.1 ± 1.7	0.240
LVMI (g/m^2^)	145.0 ± 4.6	147.2 ± 11.5	144.6 ± 5.1	0.837
Vmax (m/s)	3.90 ± 0.07	3.42 ± 0.13	3.97 ± 0.08	0.008**
MPG (mmHg)	36.6 ± 1.5	28.2 ± 2.4	37.9 ± 1.7	0.028*
AVA (cm^2^)	0.75 ± 0.02	0.70 ± 0.05	0.76 ± 0.02	0.288
Plasma creatinine (mg/dl)	1.24 ± 0.07	1.44 ± 0.23	1.20 ± 0.08	0.269
6MWT (m)	222.6 ± 13.1	124.0 ± 29.1	236.9 ± 13.6	0.003**
STS score	5.25 ± 0.65	6.27 ± 1.45	5.07 ± 0.72	0.514
EuroSCORE II	6.63 ± 0.80	9.98 ± 2.11	6.06 ± 0.85	0.084
NYHA-class	2.78 ± 0.08	3.21 ± 0.11	2.71 ± 0.08	0.001**
AF, n (%)	41	10 (71)	31 (36)	0.013*
CAD, n (%)	70	10 (71)	60 (70)	0.900
Prior CABG, n (%)	12	2 (14)	10 (12)	0.777
Prior MI, n (%)	18	4 (29)	14 (16)	0.267
PAD, n (%)	24	6 (43)	18 (21)	0.075
DM, n (%)	39	12 (86)	27 (31)	0.0001***
CRD, n (%)	26	4 (29)	22 (26)	0.813

All continuous variables are presented as mean ± SEM. All categorical variables are presented as absolute and relative frequency

*p < 0.05, ** *p* < 0.01, *** *p* < 0.001

*6MWT* 6-min walking test; *AF* Atrial fibrillation; *AVA* Aortic valve area; *BMI* Body mass index; *CABG* Coronary artery bypass graft; *Cardiac death* Patients who died in the period of observation due to cardiac reasons, cardiac reason was defined according to the VARC-2 definition; *CAD* Coronary artery disease; *CRD* Chronic respiratory disease (chronic obstructive pulmonary disease or lung fibrosis); *DM* Diabetes mellitus; *EuroSCORE* European System for Cardiac Operative Risk Evaluation; *IRX3* Iroquois homeobox 3; *LAVI* Left atrial volume index; *LVEDD* Left ventricular end-diastolic diameter normalized to BSA; *LVEF* Left ventricular ejection fraction; *LVESD* Left ventricular end-systolic diameter normalized to BSA; *LVMI* Left ventricular mass index; *MI* Myocardial infarction; *MPG* Mean pressure gradient; No cardiac death, patients who did not die in the period of observation due to cardiac reasons; *NYHA* New York heart association; *PAD* Peripheral artery disease; *STS* Society of Thoracic Surgeons; *Vmax* Aortic jet velocity. *Group comparison* Unpaired Student’s t-test for continuous variables and Pearson’s Chi^2^-test for categorical variables

#### Comparison of baseline characteristics depending on the amount of *IRX3* promoter methylation

The division of the study cohort by the *IRX3* promoter methylation led to a group of participants with a promoter methylation above (IRX3^5mc−high^) and the other group below (IRX3^5mc−low^) the median (median = 1.39%). No statistical difference between both groups could be detected for the basic characteristic parameters, such as age, body mass index (BMI) or sex, and all other baseline parameters showed no significant difference (Table 3).

**Table 3:** Baseline characteristics depending on *IRX3* methylation level

	Total cohort (*n* = 100)	IRX3^5mc−low^ (*n* = 50)	IRX3^5mc−high^ (*n* = 50)	*p* value
Age (years)	78.4 ± 0.6	77.6 ± 0.9	79.2 ± 0.9	0.208
Female sex, n (%)	41	22 (44)	19 (38)	0.542
BMI (kg/m^2^)	28.1 ± 0.5	28.2 ± 0.8	28.1 ± 0.7	0.955
Cardiac fibrosis (%)	17.8 ± 2.2	21.6 ± 3.7	15.0 ± 2.6	0.136
LVEF (%)	51.8 ± 1.6	48.9 ± 2.7	53.7 ± 1.9	0.140
LAVI (ml/m^2^)	49.9 ± 1.6	51.7 ± 2.5	48.0 ± 2.2	0.265
LVEDD (mm/m^2^)	24.5 ± 0.4	25.0 ± 0.6	24.0 ± 0.6	0.263
LVESD (mm/m^2^)	18.7 ± 0.5	19.6 ± 0.8	18.0 ± 0.7	0.137
LVMI (g/m^2^)	145.0 ± 4.6	149.1 ± 7.8	142.0 ± 5.7	0.454
Vmax (m/s)	3.90 ± 0.07	3.90 ± 0.10	3.90 ± 0.11	0.936
MPG (mmHg)	36.6 ± 1.5	36.2 ± 1.9	36.9 ± 2.4	0.811
AVA (cm^2^)	0.75 ± 0.02	0.77 ± 0.02	0.73 ± 0.03	0.245
Plasma creatinine (mg/dl)	1.24 ± 0.07	1.26 ± 0.09	1.22 ± 0.12	0.791
6MWT (m)	222.6 ± 13.1	229.1 ± 18.7	216.2 ± 18.4	0.624
STS score	5.25 ± 0.65	5.79 ± 0.61	4.66 ± 1.17	0.384
EuroSCORE II	6.63 ± 0.80	6.57 ± 0.81	6.71 ± 1.41	0.932
NYHA-class	2.78 ± 0.08	2.90 ± 0.10	2.66 ± 0.11	0.115
AF, n (%)	41	22 (44)	19 (38)	0.542
CAD, n (%)	70	33 (66)	37 (74)	0.383
Prior CABG, n (%)	12	5 (10)	7 (14)	0.538
Prior MI, n (%)	18	7 (14)	11 (22)	0.298
PAD, n (%)	24	14 (28)	10 (20)	0.349
DM, n (%)	39	19 (38)	20 (40)	0.838
CRD, n (%)	26	15 (30)	11 (22)	0.362

All continuous variables are presented as mean ± SEM. All categorical variables are presented as absolute and relative frequency

*p < 0.05

*6MWT* 6-min walking test; *AF* Atrial fibrillation; *AVA* Aortic valve area; *BMI* Body mass index; CABG: coronary artery bypass graft; CAD: coronary artery disease; CRD: chronic respiratory disease (chronic obstructive pulmonary disease or lung fibrosis); DM: diabetes mellitus; EuroSCORE: European System for Cardiac Operative Risk Evaluation; *IRX3*^*5mc−high*^: *IRX3* methylation level above median (*IRX3* methylation level ≥ 1.39%); *IRX3*^*5mc−low*^: *IRX3* methylation level below median (*IRX3* methylation level < 1.39%); LAVI: left atrial volume index; LVEDD: left ventricular end-diastolic diameter normalized to BSA; LVEF: left ventricular ejection fraction; LVESD: left ventricular end-systolic diameter normalized to BSA; LVMI: left ventricular mass index; MI: myocardial infarction; MPG: mean pressure gradient; NYHA: New York heart association; PAD: peripheral artery disease; STS: Society of Thoracic Surgeons; Vmax: aortic jet velocity. Group comparison: Unpaired Student’s t-test for continuous variables and Pearson’s Chi^2^-test for categorical variables

#### Correlation analysis between *IRX3* promoter methylation and distinguished clinical parameters

**Fig. 3:**
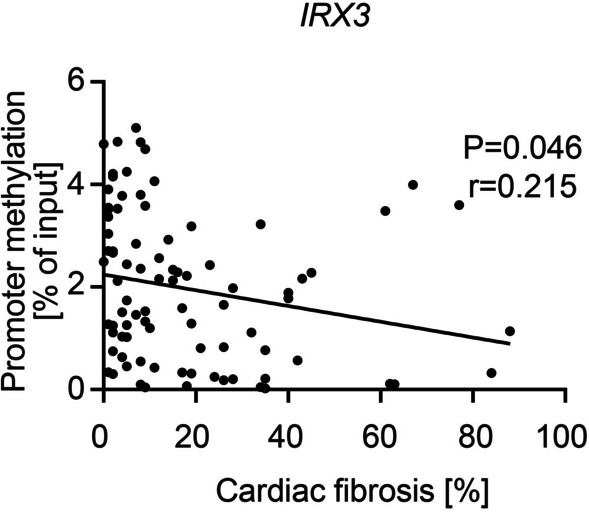
Significant inverse correlation between circulating *IRX3* promoter methylation level and cardiac fibrosis. The circulating *IRX3* promoter methylation and the amount of cardiac fibrosis correlate inversely on a significant level but with a relatively weak correlation coefficient (*r* = 0.215). Each dot representing one individual (*n* = 100), regression shown as simple linear regression. Pearson’s correlation coefficient (Pearson’s r) and *p* value were calculated

As basic parameters, age and BMI were included into the correlation analysis. In addition, cardiac fibrosis due to its newly identified role in AS was also included [8]. Interestingly, a significant but very weak inverse correlation between the promoter methylation of *IRX3* and the amount of cardiac fibrosis was detected (Table 4, Fig. 3).

**Table 4:** Correlations between circulating *IRX3* promoter methylation level and distinguished clinical parameters

	IRX3 promoter methylation	
	Pearson’s r	*p* value
Age	0.107	0.290
BMI	0.088	0.381
Cardiac fibrosis	− 0.215	0.046*

*BMI* Body mass index. Correlation analyses were performed by determining Pearson’s correlation coefficient (Pearson’s r) and P-value, respectively. * *p* < 0.05

### Survival analysis

**Fig. 4:**
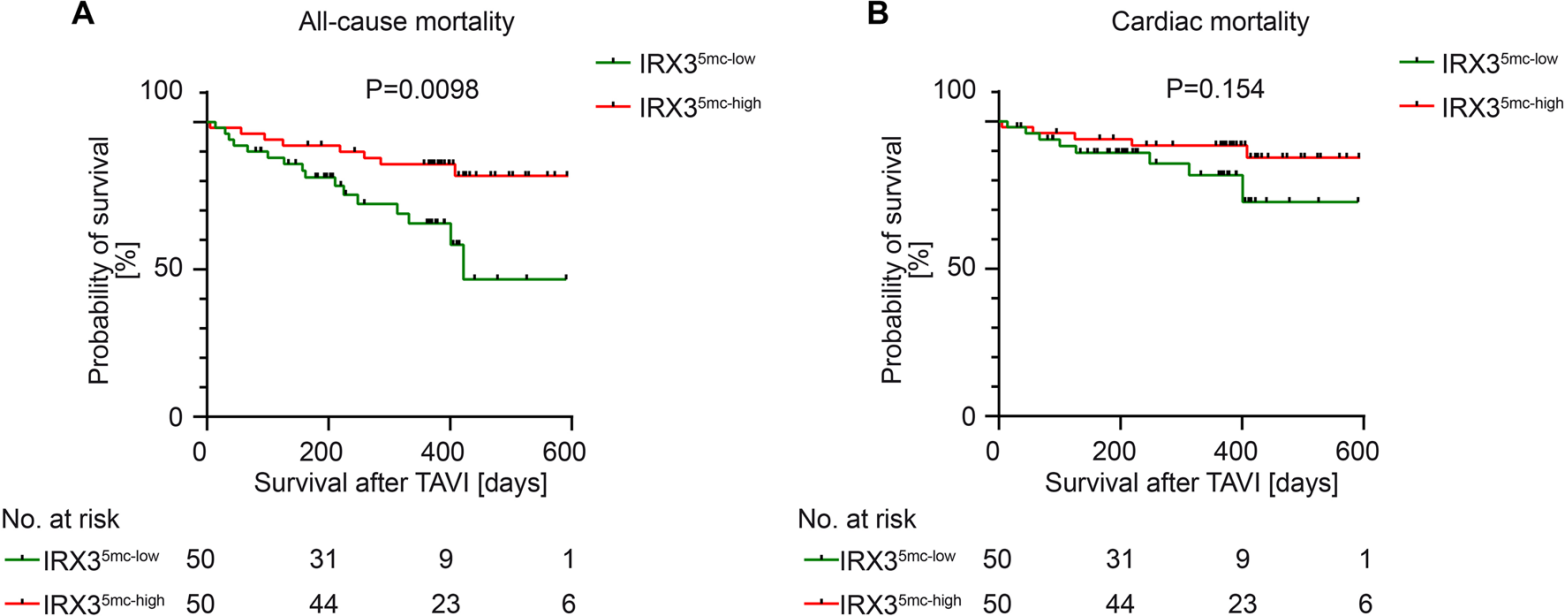
Survival analysis depending on circulating *IRX3* promoter methylation level. Kaplan–Meier curves showing all-cause (**A**) and cardiac (**B**) mortality for the first 600 days after TAVI in patients with circulating *IRX3* promoter methylation below (green) and above (red) the median. **A** Patients with promoter methylation above the median show a better survival and outcome after TAVI as compared to those with promoter methylation below the median for all-cause mortality. **B** Both groups of promoter methylation above and promoter methylation below the median show no significant difference for cardiac mortality after TAVI

To assess the correlation between *IRX3* promoter methylation levels and patient mortality, two different analyses were performed: 1. univariable survival analyses on previously defined two groups (IRX3^5mc−high^, IRX3^5mc−low^) and 2. multivariable survival analyses. Both groups were compared based on all-cause (Fig. 4A) and cardiac mortality (Fig. 4B). All-cause mortality was clearly predicted by a low *IRX3* promoter methylation (*p* = 0.0098). For cardiac mortality, no significant difference could be detected.

**Fig. 5:**
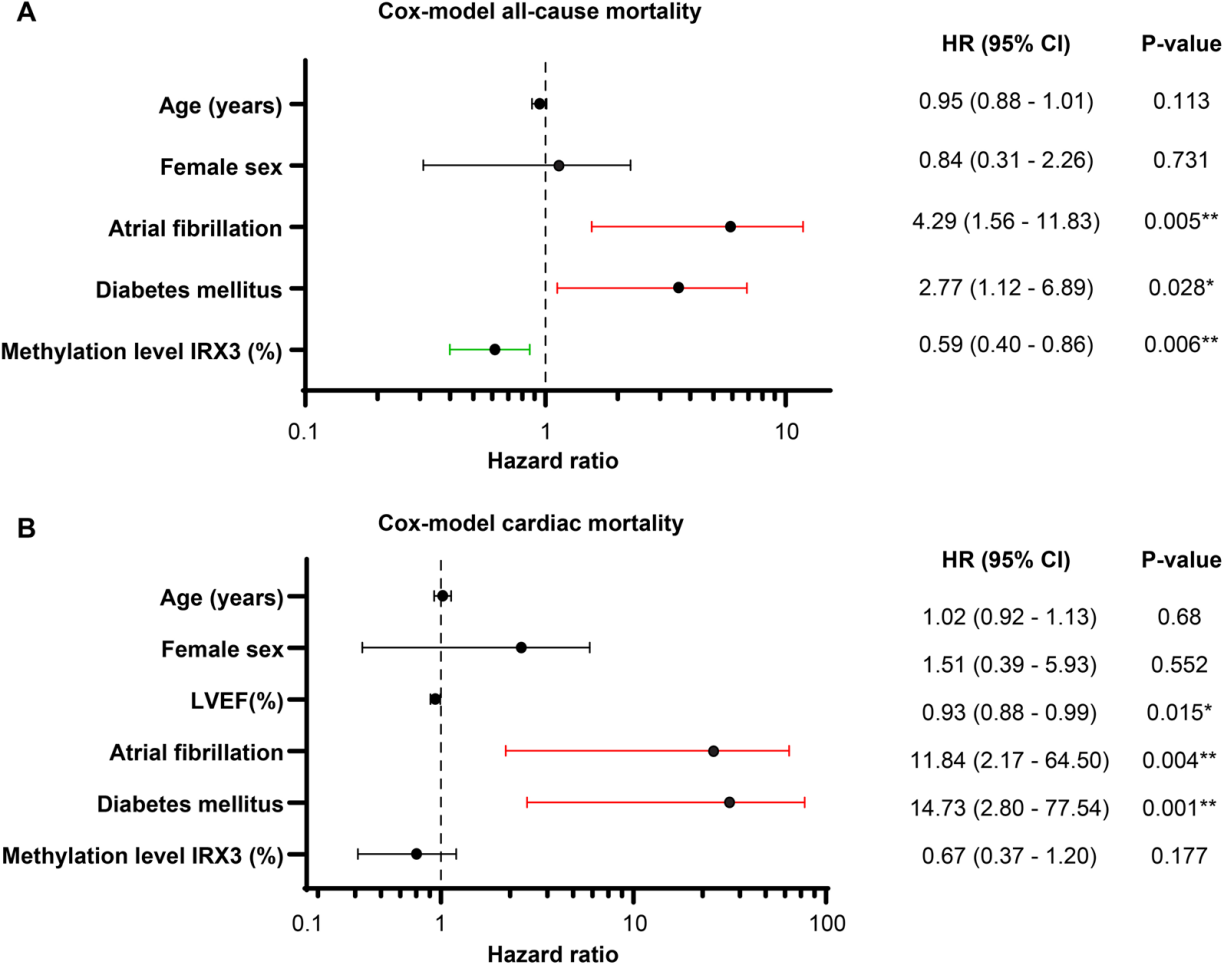
Multivariable Cox-regression model for all-cause and cardiac mortality after TAVI. The final multivariable Cox-regression models for all-cause (**A**) and cardiac (**B**) mortality. **A** The final model included AF and DM as independent predictors of all-cause mortality, whereas an increase in *IRX3* promoter methylation level was protective. **B** The final model included AF and DM as independent predictors of cardiac mortality, whereas LVEF was protective. *IRX3* promoter methylation level showed no influence on cardiac mortality. CI: confidence interval; HR: hazard ratio; LVEF: left ventricular ejection fraction. * *p* < 0.05, ** *p* < 0.01

For the multivariable survival analyses, multivariable Cox-regression was performed. As a first block of the model, age and sex were taken as basic demographic parameters and *IRX3* promoter methylation was added as the variable of interest. In the next block, eleven relevant clinical parameters deemed to be related to outcomes (Cardiac fibrosis, LVEF, Vmax, MPG, AVA, Plasma creatinine, NYHA-class, AF, CAD, DM, CRD) were automatically added stepwise using forward covariate selection based on likelihood ratio, until the most suitable model was reached. To compare the different models, − 2 log likelihood was used as a metric for evaluation. The final model (− 2 log likelihood = 140.81, Chi-square = 23.98, *p* < 0.001***) included AF and DM as statistically significant parameters for the prediction of all-cause mortality and in line with the results from Kaplan–Meier analysis and even though many clinical parameters were included for the analysis, *IRX3* promoter methylation was an independent predictor for all-cause mortality as well (Fig. 5A, HR = 0.59, CI = 0.40–0.86, *p* = 0.006).

The model for cardiac mortality was designed and assessed in the same way as for all-cause mortality. The final model for cardiac mortality (− 2 log likelihood = 70.63, Chi-square = 34.16, *p* < 0.001***) included LVEF, AF and DM as statistically significant parameters for the prediction of cardiac mortality. *IRX3* promoter methylation showed no significant influence on cardiac mortality (Fig. 5B, HR = 0.67, CI = 0.37–1.20, *p* = 0.177). This contrasts the findings from the Cox-regression model on all-cause mortality but is in line with the results from Kaplan–Meier analysis for cardiac mortality.

### Discussion

The present study was designed to examine the potential connection between the circulating *IRX3* promoter methylation and survival after TAVI. The key findings are: (1) Circulating *IRX3* promoter methylation below the median independently predicts all-cause mortality in both univariable and multivariable analyses but not cardiac mortality after TAVI. (2) Patients that died from all-cause death had significantly lower *IRX3* promoter methylation, but those that died from cardiovascular causes had no difference in *IRX3* promoter methylation. (3) The expression of *IRX3* was higher in myocardial biopsies from patients with low levels of circulating *IRX3* promoter methylation, whereas there was no correlation between IRX3 positive area in the heart and cardiac fibrosis. (4) Promoter hypermethylation decreases *IRX3* gene expression in vitro. (5) There is a significant but very weak inverse correlation between circulating *IRX3* promoter methylation and cardiac fibrosis.

This study demonstrates a strong influence of promoter hypermethylation as an epigenetic regulatory mechanism on gene expression of *IRX3*. Many studies showed a link between promoter hypermethylation and reduction of gene expression, which match our findings [31, 32]. Evidence for the reflection of gene expression by circulating methylated DNA is rarely available, due to its release from different organs by distinctive mechanisms [18, 19]. We are aware that the origin of the circulating methylated DNA in our clinical samples is unspecified. However, cardiomyocyte specific circulating DNA has recently been identified and has shown to be elevated in patients with myocardial damage [33]. Differently methylated regions of DNA were also reported in aortic and mitral valve tissue, and the linked genes are associated with (patho-) physiological pathways [34]. Results from our immunohistochemical staining experiment suggested an inverse correlation between circulating *IRX3* promoter methylation and gene expression in myocardial biopsies. Therefore, the connection between circulating *IRX3* promoter methylation and *IRX3* expression in the heart can be assumed. However, further studies on the release and origin of circulating DNA, especially in cardiac diseases, are needed to further investigate and prove this correlation.

Further, many independent studies described the harmful impact of cardiac fibrosis in patients with AS, where increased cardiac fibrosis is associated with a worse clinical outcome [8, 35–38]. Our data are in line with this observation, as the amount of cardiac fibrosis was significantly increased in the group of cardiac deaths. Additionally, correlation analysis between cardiac fibrosis and circulating *IRX3* promoter methylation showed a weak but significant inverse correlation, as high levels of cardiac fibrosis were associated with less *IRX3* promoter methylation. As this is related to a higher genetic expression, a pro-fibrotic influence of high *IRX3* expression levels might be possible but had not been discussed in the literature before. In contrast, however, there was no correlation between IRX3 positive area in the biopsies and cardiac fibrosis, so the validity of these observations remains limited. Again, further research on the function of IRX3 in the mammalian heart is needed to uncover a possible connection.

In the present study, univariable and multivariable survival analyses revealed an association between circulating *IRX3* promoter methylation and all-cause mortality. Surprisingly, for cardiac mortality, no effect of *IRX3* promoter methylation on survival was shown in either univariable or multivariable analysis. A possible explanation for the missing influence on cardiac mortality is the low number of cardiac deaths (*n* = 14) in comparison with all-cause deaths (*n* = 25). Additionally, the number of co-morbidities in the study cohort was very high. As *IRX3* promoter methylation was only connected to all-cause and not to cardiac mortality, it may be related to factors other than cardiac damage. Since the co-morbidities were not significantly different between the groups of high and low circulating *IRX3* promoter methylation, our results suggest that levels of circulating methylation *IRX3* promoter fragments can add prognostic value beyond simply indicating co-morbidities. Also in the multivariable Cox-regression model for all-cause mortality, *IRX3* promoter methylation remained statistically significant along with diabetes and atrial fibrillation, whereas the other clinical parameters did not.

Additionally, there was no effect of CAD on both cardiac and all-cause mortality, although the influence has already been shown previously [39]. The prevalence of CAD in our cohort was very high (70%), so due to methodical reasons it is likely that it does not show an influence on survival as an important factor. Since we do not have additional information on complexity and severity of CAD, no further statements can be made about the influence of CAD in our cohort.

As nowadays clinical guidelines recommend intervention for every patient with symptomatic severe AS but not for asymptomatic patients, intervention in those patients is performed only in exceptional cases or clinical trials [3]. A recent study showed that early surgical valve replacement in asymptomatic patients compared to standard conservative treatment improved the outcome during follow-up [40]. However, this study had several limitations such as small size of the study cohort and relatively young age (mean = 64 years), so no new principles for treatment were derived from it. The latest guidelines for AS management recommend intervention in asymptomatic patients if the procedural risk is low and BNP levels are elevated more than three times normal range, but usually procedural risk in TAVI patients is elevated due to their amount of co-morbidities, so this might not be applicable to our cohort [3]. Risk scores, as the EuroSCORE II, which was designed for general cardiac surgery, were tested in TAVI cohorts and especially the EuroSCORE II predicted 30-day mortality after TAVI, but only symptomatic patients were included in this study and the authors criticized the lack of gaining additional information by using this score [41]. Further biomarkers which are well established for other cardiac diseases, as MI or heart failure, were examined in patients with severe AS undergoing surgical aortic valve replacement (SAVR). Elevated levels of NT-proBNP (N-terminal pro b-type natriuretic peptide) combined with hsTropT (high-sensitivity troponin T) as well as CRP (C-reactive protein) were associated with a worse outcome after SAVR [42]. Multiple studies investigated novel potential biomarkers, i.e., malondialdehyde, Galectin-3 or specific circulating micro-RNAs, with promising results, but mostly in small cohorts without randomization [43–45]. Epigenetic alterations which could potentially be used as biomarkers were also described for other cardiovascular diseases, e.g., methylated DNA of CD4 + cells in pulmonary arterial hypertension or DNA methylation profile of human leukocyte antigen-G in CAD [46, 47]. Our findings add a novel candidate into this emerging and fast-developing field, as low circulating *IRX3* promoter methylation predicted a worse outcome after TAVI, and thus might aid as a biomarker for identify AS patients at higher risk and to monitor these patients more closely, even though the increased risk of mortality may not be due to cardiac factors. Therefore, our biomarker should be also studied in other cohorts suffering from diseases outside of the cardiovascular field, to further evaluate its association with mortality.

To target the pathophysiology of AS and slow down or even stop its development by specific drugs is the most desirable method to end the enormous burden caused by this disease. Although many attempts have been made in this regard, no specific pharmaceutical therapy is available today [48]. Previous studies revealed a harmful impact of DNA methylation on disease progression due to the suppression of fibrosis suppressor or tumor suppressor genes [49–51]. Since the methylation of DNA is a reversible process, it is a highly promising approach to target therapeutically [52]. Therefore, previous research has focused on the use of demethylating agents as therapeutic approaches, such as 5-azacytidine in cardiac hypertrophy and fibrosis, or hydralazine in chronic kidney disease [50, 51]. In contrast, in our study, methylation of *IRX3* appears to have a beneficial effect on survival. In case future studies demonstrate a functionally beneficial role of methylated *IRX3*, this would raise the possibility of a specifically methylating epidrug. Certainly, it is still too early to conclude from these results on clinical application. In order to identify if *IRX3* could serve as a therapeutic target or diagnostic marker for future clinical purpose, further studies on its molecular function both inside and outside of the heart are needed.

### Conclusions

This study found that low levels of *IRX3* methylation in the blood of AS patients at the time of TAVI are associated with poorer survival after TAVI and at least partially reflect higher myocardial IRX3 expression. Therefore, circulating methylated *IRX3* might aid as a potential biomarker to help guide both pre-TAVI patient selection and post-TAVI monitoring.

### Limitations

Firstly, our study is not a randomized study. The decision to perform TAVI instead of SAVR in patients with AS is biased by age, co-morbidities and the patient’s request. This selection bias resulted in a study cohort of elderly patients with a high number of co-morbidities, thus influencing all-cause and cardiac mortality. Secondly, it was a single-center study with only one hundred participants. Due to the limited size, the validity of the findings for the population is limited. Preferably, to increase the power and validity of the findings, another study with a higher number of participants is needed. In addition, not all patients completed 2-years follow-up, which could lead to the false assumption that subjects are still alive even though they have already died. In order not to allow this false assumption, the date of last visit was assessed for these subjects and Kaplan–Meier analysis and Cox-regression take these censored subjects into account methodically. Lastly, a relatively small number of samples was used for IHC staining of IRX3, and conclusion from this small number may be limited.

## Methods

### Study cohort

Between January 2017 and May 2019, 100 patients underwent transfemoral TAVI and were enrolled to the study. The university’s ethics committee approved the study and informed consent was obtained from each patient. All procedures were performed conform to the World Medical Association Declaration of Helsinki. All patients had severe AS. Several patients also had other valvular heart disease but those were not severe. All patients were symptomatic and all had dyspnea. Rates of angina, however, were not assessed. At baseline, transthoracic (TTE) and transesophageal (TEE) echocardiography were performed, and 6-min walking test (6MWT), STS score, EuroSCORE II and New York Heart Association (NYHA) status were assessed, and plasma creatinine levels were measured. Follow-up visits were scheduled after six months, one year and two years. The primary endpoint was defined by cardiovascular mortality according to the VARC-2 definition [53] and the secondary endpoint by all-cause mortality.

### Human myocardial tissue

During TAVI, cardiac biopsies were harvested from the basal anteroseptum of the left ventricle in 88 out of the 100 patients of our cohort. The samples were placed in 10% paraformaldehyde and embedded in paraffin. The amount of cardiac fibrosis was assessed using the software CellSens (Olympus, Shinjuku, Japan), where fibrotic area was defined as blue area in Masson’s trichrome staining (MTS).

### DNA extraction and MeDIP

Evidence can be found for rapid clearance of circulating cell-free DNA from the blood and it has been further described that almost all extracellular nucleic acids in individuals without malignancies are bound at the surface of blood cells including leukocytes [54–56]. Additionally, distinguished DNA methylation sites in patients with heart failure which are conserved between cardiac tissue and whole-peripheral blood have recently been identified [57]. Therefore, we decided to investigate the peripheral methylation in the cellular compartment of whole blood containing leukocytes (buffy coat) instead of plasma. Human circulating DNA was isolated from the buffy coat layer of anticoagulated EDTA (ethylenediaminetetraacetic acid) blood samples using the DNeasy Blood & Tissue Kit (Qiagen, Venlo, the Netherlands) according to the manufacturer’s protocol. For maximum DNA yield, 100 µL buffy coat were taken. In the next stage, Methylated DNA immunoprecipitation (MeDIP) was performed using the Methylamp Methylated DNA Capture Kit (Epigentek, Farmingdale, NY, USA). In the first step, 1.2 µg of isolated DNA was sheared by sonication and then denatured to obtain single-stranded DNA. One microgram of the provided DNA was then placed inside each antibody coated well and further treated as described in the manufacturer’s protocol. An input vial was performed for each sample to act as a loading control.

### Real-time quantitative PCR (qPCR)

To assess the methylated DNA amplification, 2 µL of immunoprecipitated DNA, 10 µL of Fast SYBR Green Master Mix (Thermo Fisher Scientific, Waltham, MA, USA) and 2 µL of primer mix (sequences listed below) were used in a final volume of 20 µL. The reaction was performed in a MicroAmp Fast 96-Well Reaction Plate (Thermo Fisher Scientific) using the StepOnePlus Real-Time PCR System (Thermo Fisher Scientific). The methylation level was quantified in comparison with the input sample using the ∆∆Ct method. Oligonucleotides sequences for MeDIP-qPCR are shown in Table 5.

**Table 5:** Primers

Usage	Sequence
MeDIP-qPCR	F: GACGTCAGACCCGAGATTTG
	R: GGTGTGTGTCGGTGTCTGTC
Cloning	F: TTAACTGCAGGGAATTTCAATGCCGCTATC
	R: TTAAAAGCTTTCGCCCTCCTCTAGTTTTCA

### Chromogenic immunohistochemical staining

Since many of the cardiac biopsies were also used for other studies, we selected ten biopsies out of the 88 biopsies randomly and solely based on abundant availability of biopsy tissue in the biobank. These patients did not share any specific characteristics. Formalin-fixed, paraffin-embedded human myocardial samples were sectioned at 3 µm, deparaffinized by xylene and rehydrated by graded ethanol-series with decreasing concentrations. Heat-induced epitope retrieval was performed with citrate buffer (Target Retrieval Solution, Citrate pH 6.1 (10x), Agilent Technologies, Santa Clara, CA, USA) for 40 min. The slides were then incubated in a 0.3% peroxidase blocking buffer (Carl Roth, Karlsruhe, Germany) for 30 min, and blocked afterward in diluted normal horse serum (Vectastain Elite ABC-HRP Kit, Vector Laboratories, Burlingame, CA, USA) for 20 min, followed by overnight incubation at 4 °C with the primary antibody (Rabbit Anti-Human IRX3, 1:50, Biorbyt, Cambridge, UK, Cat-No. orb127001). Next, the slides were incubated with the biotinylated secondary anti-rabbit antibody (1:50, Vectastain Elite ABC-HRP Kit, Vector Laboratories) for 30 min. Afterward, the samples were incubated in the avidin–biotin conjugate (ABC-solution, Vectastain Elite ABC-HRP Kit, Vector Laboratories) for 30 min, followed by incubation with AEC Substrate-Chromogen (Agilent Technologies, Santa Clara, CA, USA) until the staining’s intensity was sufficiently. Nuclear staining was performed with acid hemalum solution according to Mayer (Carl Roth). Microscopic pictures were taken using the Olympus microscope BX43 and CellSens software. Six representative visual fields were selected randomly and the intensity of staining was assessed using the FIJI software [58]. The amount of IRX3 was defined as red colored area in relation to total image area.

### Cloning and in vitro methylation

*IRX3* promoter sequence was amplified using the Phusion High-Fidelity PCR kit (New England Biolabs, Ipswich, MA, USA) according to the manufacturer’s protocol with the primer sequences listed in Table 5 and an amount of 100 ng genomic DNA. Afterward, electrophoresis was performed on a 1.2% agarose gel and DNA was extracted from the gel using the QIAquick Gel Extraction kit (Qiagen) according to the manufacturer’s protocol. Further, the pCpGL-basic vector and *IRX3*-promoter insert were digested with *Hin*dIII/*Pst*I restriction enzymes. Vector and insert were ligated using T4 DNA ligase (Promega, Fitchburg, MA, USA). To amplify the construct, transformation was performed using the One Shot PIR1 Chemically Competent *E. coli* (Thermo Fisher Scientific). The ligated DNA was inserted using the heat shock method and selected by the usage of the antibiotic zeocin (25 µg/mL). Miniprep was performed using the Zyppy Plasmid Miniprep kit (Zymo Research, Freiburg, Germany) as described in the manufacturer’s protocol. Transformation efficacy was confirmed by gel electrophoresis and sequencing. For enhanced plasmid yield, Midiprep was performed using the HiSpeed Plasmid Midi kit (Qiagen) according to the manufacturer’s protocol. The pCpGL-*IRX3* plasmid was then methylated in vitro using the CpG (cytosine-phosphate-guanine) Methylase (M. Sssl) kit (Zymo Research) according to the manufacturer’s protocol by using 3 µg of isolated plasmid DNA in a total reaction volume of 60 µL. The methylated DNA was isolated using the DNA Clean & Concentrator-5 kit (Zymo Research) as described in the manufacturer’s protocol.

### Cell culture and luciferase assay

Human Embryonic Kidney cells (HEK-293, ATCC, Manassas, VA, USA) were maintained in DMEM (Dulbecco's modified eagle medium) low-glucose medium (Thermo Fisher Scientific) supplemented with 10% FBS (fetal bovine serum) (Thermo Fisher Scientific) and 1% penicillin–streptomycin (Sigma-Aldrich, St. Louis, MO, USA). For transfection, 20,000 cells/well were seeded into each well of a 24-well plate. Transfection was performed using Lipofectamine 3000 (Thermo Fisher Scientific) according to the manufacturer’s protocol. 1 µg of plasmid DNA, 1 µL of Lipofectamine 3000, and 2 µL of P3000 Enhancer Reagent were mixed with Opti-MEM I medium (Thermo Fisher Scientific) and added to the cells. Cells were harvested 48 h after transfection and used for luciferase assay by using the Dual-Luciferase Reporter Assay System kit (Promega, Fitchburg, MA, USA) according to the manufacturer's instructions, but only measuring the firefly luciferase activity. The lysate was transferred into a Lumix 96-multiwell plate (Sarstedt, Nümbrecht, Germany) and 50 µL of Luciferase Assay Reagent II were placed into each well. Luciferase activity was assessed by the Fluorescence Microplate Reader Synergy Mx (Biotek Instruments, Winooski, VT, USA). The reduction of luciferase activity was calculated compared to the unmethylated control and normalized to the empty pCpGL-basic vector.

### Statistics

All data were presented as mean ± standard error mean (SEM). P-value less or equal to 0.05 was defined as statistically significant. In vitro experiments were carried out with three or more biological replicates for all experiments. Statistical analysis was performed using GraphPad Prism 8 software (GraphPad Software, La Jolla, CA, USA). Unpaired Student’s t-test was used for single comparison and one-way ANOVA for comparing multiple groups. Human data were analyzed using the SPSS Statistics program (IBM, Armonk, NY, USA). A median split by *IRX3* promoter methylation and by survival was performed to further characterize the study cohort. Continuous variables were compared using unpaired Student’s t-test. Categorical variables were presented as absolute numbers and percentage and compared by Pearson’s Chi^2^-test. For direct correlation analysis, Pearson’s correlation coefficient was determined. Survival was analyzed using Kaplan–Meier plot and logrank test for univariable analysis in the period up to 600 days after TAVI. Multivariable models were calculated using Cox-regression model. As a first block of the model, age and sex were taken as basic demographic parameters and *IRX3* promoter methylation was added as the variable of interest. In the next block, eleven relevant clinical parameters deemed to be related to outcomes (Cardiac fibrosis, LVEF, Vmax, MPG, AVA, Plasma creatinine, NYHA-class, AF, CAD, DM, CRD) were automatically added stepwise using forward covariate selection based on likelihood ratio, until the most suitable model was reached. To compare the different models, − 2 log likelihood was used as a metric for evaluation. No Bonferroni correction was performed due to the study design as a pilot study.

## Data Availability

The datasets used and/or analyzed during the current study are available from the corresponding author on reasonable request.
